# Postoperative myocardial injury in a patient with left ureteric stone and asymptomatic COVID-19 disease

**DOI:** 10.11604/pamj.2020.36.170.23882

**Published:** 2020-07-10

**Authors:** Nadeem Kassam, Omar Aziz, Ally Zain Ismail, Rodgers Swai, Samina Somji, Robert Mvungi, Mustaafa Bapumia, Aliakber Zehri, Salim Surani

**Affiliations:** 1 Department of Internal medicine, Aga Khan University, Medical college, East Africa,; 2 Department of Internal medicine, Aga Khan Hospital, Dar-es-salaam, Tanzania; 3 Department of Surgery, Aga Khan University, Medical college, East Africa,; 4 Department of Intensive care, Aga khan Hospital, Dar-es-salaam, Tanzania,; 5 Department of Cardiology, Aga Khan Hospital, Dar-es-salaam, Tanzania,; 6 Department of Internal Medicine, Texas A&M Health Science Center, Bryan, USA

**Keywords:** Coronavirus disease 2019 (COVID-19), severe acute respiratory syndrome coronavirus 2, myocardial infarction, ureteric calculi

## Abstract

Coronavirus disease 2019 (COVID-19) is an infectious disease caused by severe acute respiratory syndrome coronavirus 2 (SARS-CoV-2). It was first identified on 8^th^December 2019 in Wuhan, Hubei, China, and has since spread globally to become an emergency of international concern. Patients infected with SARS-CoV-2 may be asymptomatic or present with symptoms ranging from mild clinical manifestations: such as fever, cough, and sore throat to moderate and severe form of the disease such as pneumonia and acute respiratory distress syndrome (ARDS). In some patients, SARS-CoV-2 can affect the heart and cause myocardial injury which is evidenced either by electrocardiographic (ECG) changes or by a rise in serum troponin level. Patients with myocardial involvement are generally at risk of developing severe illness and tend to have a poor outcome. We hereby present a case of a hypertensive male patient with undiagnosed, asymptomatic COVID-19, who underwent an emergency urologic procedure for ureteric calculi. He eventually sustained a postoperative myocardial injury resulting in his demise. This case highlights the importance of detailed preoperative assessment and anticipation of complications during this global pandemic.

## Introduction

SARS-CoV-2 has spread across the world in a very short period, posing an extraordinary challenge. According to the situation report published by the World Health Organization, as of April 30^th^, 2020, it has affected more than 3 million people worldwide with more than 200,000 deaths. Patients with COVID-19 who have underlying cardiovascular disease and diabetes are at added risk of poor outcomes. Myocardial injury has been considered as an essential pathogenic feature of COVID-19 however the precise mechanism is not completely clear. It has been hypothesized that it generally results either from direct damage of myocytes or systemic inflammation resulting in an interferon-mediated immune response. Additionally, the cytokine storm has also been considered to trigger stable coronary artery plaque which subsequently results in myocardial injury [1]. Therefore, it is expected that elderly patients with cardiovascular risk factors are predisposed to adverse consequences of the disease compared to younger individuals.

## Patient and observation

**Figure 1:**
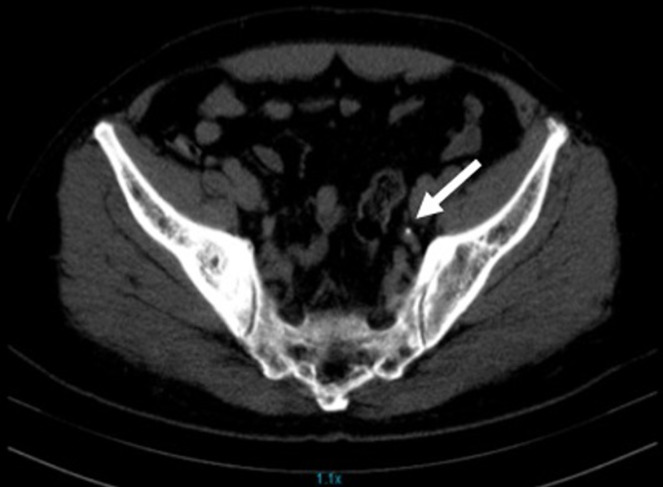
computed tomography of kidneys, ureters, and bladder revealed an obstructive left ureteric calculus at the vesicoureteric junction (VUJ) measuring 4.7 mm with no obvious fat stranding

**Figure 2:**
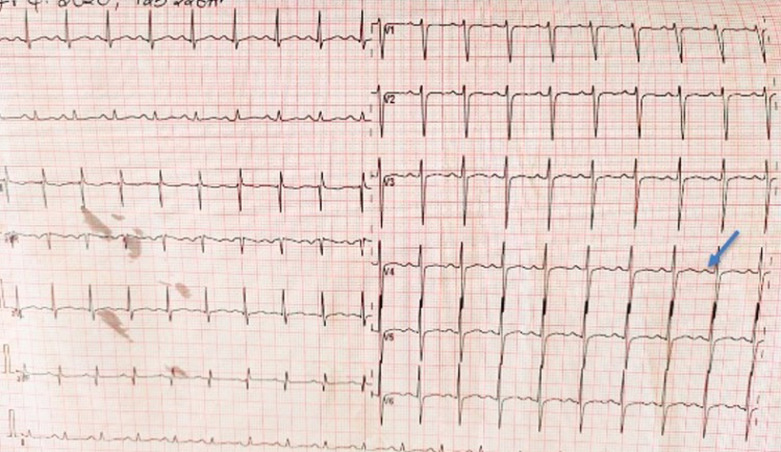
ECG showing normal sinus rhythm with sinus tachycardia

A 60-year-old male with a history of hypertension presented with an acute, severe, left lumbar pain, radiating to the groin, associated with nausea and dull pain on micturition. He denied any history of fever or hematuria. He reported upper respiratory tract symptoms, including cough and chest pain for 10 days before admission which had slowly resolved. The patient denied having been in close contact with a COVID-19 patient. On examination, he was alert and oriented with stable vital signs. An abdominal examination revealed a tender left lumbar region. The rest of the physical examination was unremarkable. Computed tomography of the kidneys, ureters, and bladder revealed an obstructive left ureteric calculus at the vesicoureteric junction (VUJ) measuring 4.7mm (Figure 1). Blood workup revealed an elevated creatinine at 134 µmol/L (59-104 µmol/L) and potassium of 6.26 mmol/L (3.3-5.1 mmol/L) suggestive of obstructive uropathy with acute kidney injury. Given the findings, the patient underwent emergency cystoscopy, left ureteroscopy, stone fragmentation, and Double J (DJ) ureteric stent insertion. The pre-operative assessment involved medication review and ECG, which was considered normal (Figure 2). Intraoperatively he was found to have significant distal left ureteric narrowing at the VUJ with a calculus proximal to the narrowing. The stone was fragmented, DJ stent was deployed, and a Foley catheter was placed to facilitate urinary drainage. In the recovery room, he was found to be desaturating at saturation of 80-85% on room air and required 8 L/min of oxygen supplementation via a face mask to maintain his oxygen saturation above 94%. He was then transferred to the ward for post-operative management and close observation. 12 hours post-transfer to the ward he was found to be tachycardic at 130 beats per minute with normal sinus rhythm (Figure 2) and blood pressure ranging from 80/50 mmHg to 90/60 mmHg. On examination, he was alert, not diaphoretic but had cool and clammy extremities. Respiratory examination revealed bilateral basal crepitations. The patient denied having lightheadedness, chest pain, difficulty in breathing, or shortness of breath.

**Figure 3:**
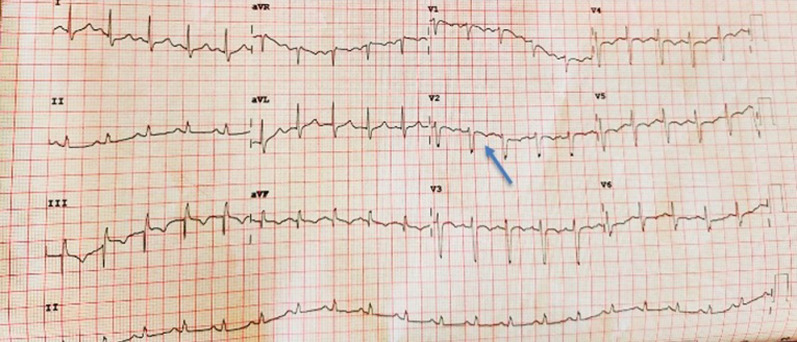
ECG showing nonspecific ST changes and T wave inversion with no ST elevation

**Figure 4:**
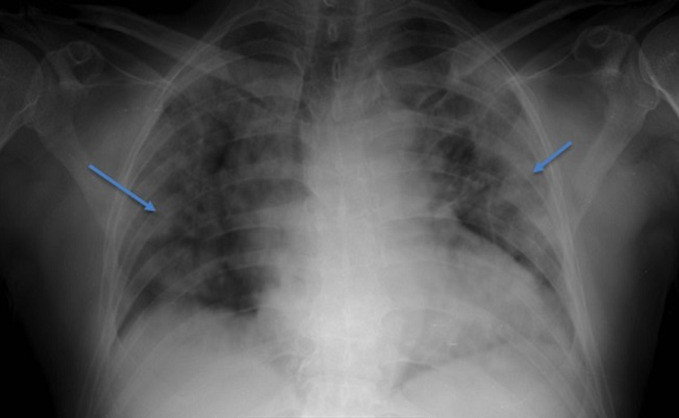
X-ray Chest showing bilateral patchy infiltrates, cardiomegaly, and unfolded aorta are seen

On workup, it was found that the patient had an abnormal ECG with ST-segment depressions and T wave inversion on the anterior (V1- V4) and inferior leads (III & aVF) (Figure 3), when compared to the preoperative ECG with markedly elevated troponin-T of 174.1 pg/ml (0-14 pg/ml). Other laboratory parameters were: Lactate dehydrogenase 494 IU/L (135-225 IU/L), Pro-BNP 375 pg/ml (0-125 pg/ml), Procalcitonin 1.67 ng/ml (0-2 ng/ml), C-Reactive Protein 312 mg/L (0.5-5 mg/L), INR 1.15, ferritin 1531 µg/l (22-322 µg/l), D-dimer 0.63 µg/ml (0-0.5 µg/ml). A portable chest x-ray revealed bilateral patchy pulmonary infiltrates with some coalescence observed in the periphery of lungs, a pattern consistent with a CO-RADS 5 classification, highly suggestive of COVID-19 (Figure 4), and was later confirmed by a nasopharyngeal swab using real-time reverse transcription-polymerase chain reaction (RT-PCR). He was transferred to an isolation intensive care unit and started on Non-ST Elevation Myocardial Infarction (NSTEMI) protocol where he received anticoagulant therapy with subcutaneous Enoxaparin 80 mg, 300 mg Clopidogrel, 600 mg Aspirin, and 80 mg Atorvastatin. Within a few hours of the patient´s transfer to critical care, his clinical condition deteriorated rapidly necessitating intravenous inotropic support. He was subsequently intubated and put on mechanical ventilation. A repeat Troponin level at 6 hours was increased to 194 pg/ml while there was further documented worsening of renal function with BUN 16.70 mmol/l and creatinine 254 µmol/L. Unfortunately, a transthoracic echocardiogram was unavailable at the time. His condition continued to deteriorate rapidly, and he went into shock and sustained a cardiac arrest. Cardiopulmonary resuscitation (CPR) was carried out according to American Cardiac Life Support (ACLS) protocol but was unsuccessful. The patient was pronounced dead after 30 minutes of CPR.

## Discussion

The COVID-19 pandemic is one of the greatest international health care challenges we face in this modern time causing global turmoil with both health and economic challenges. So far, the focus of COVID-19 has primarily been the respiratory system. Extrapulmonary manifestation is still somewhat unfamiliar and variable, nevertheless, recent studies have recognized the role of cardiovascular disease as a risk factor of the severity of the infection [2]. Cardiovascular manifestations are not unique to this particular pandemic, SARS-CoV-2 appears to have similar pathophysiology of myocardial injury to the coronaviruses responsible for previous Severe Acute Respiratory Syndrome coronavirus (SARS) and the Middle East Respiratory Syndrome coronavirus (MERS-CoV) outbreak [3]. Myocarditis is a reflection of myocardial damage, out of the several mechanisms; direct myocardial injury due to fulminant viral myocarditis has been considered the most common [3]. This phenomenon results in circulatory and respiratory collapse, as observed in our patient and carries a very high in-hospital mortality of above 50% [4].

Diagnosis of myocarditis is a diagnostic dilemma it is practically a clinical concept and very few cases have been confirmed radiologically. In a case published by Inciardi *et al*.[5] the diagnosis of myocarditis was established with cardiac MRI which revealed biventricular oedema and gadolinium enhancement, a diagnostic tool currently not available in our setting. It is unclear what the precise precipitating event of our patient´s sudden cardiac arrest was, though malignant arrhythmias have also been well noted in patients with COVID-19 who are admitted to intensive care units (ICU). It has been assumed that the arrhythmias are likely to be attributable to hypoxia, metabolic disarray, or neurohormonal stress in the background of COVID-19 infection [6]. In terms of disease outcome, a cross-sectional study by Chen *et al*.[7] revealed: elevated N-terminal pro brain natriuretic peptide (NT-proBNP), cardiac troponin I (cTnI), and C- Reactive protein (CRP) was collectively suggestive of myocardial injury and inflammation that correlated with critical illness and poor outcomes. In the same study, it was also noted elderly patients with a history of hypertension and elevated serum creatinine were prone to poor prognosis as witnessed in our patient.

## Conclusion

This case also highlights the need for detailed and standardized pre-operative assessment during the current COVID-19 pandemic, especially for asymptomatic patients. Comprehensive assessments will not only help to control disease transmission amongst staff but also prepare medics better for possible anticipation of complications. Since patients affected by SARS-CoV-2 can present with no or varying symptoms, it is logical to label or manage everyone as COVID-19 suspect until results are confirmed. It is becoming more obvious that many cases are pre-symptomatic or asymptomatic and the prevalence of myocardial injury in various populations is not yet known, as Troponin and ECGs are generally reserved for hospitalized patients.
